# Supplementary material for: Gene Editing of the Catfish Gonadotropin-Releasing Hormone Gene and Hormone Therapy to Control the Reproduction in Channel Catfish, Ictalurus punctatus
Source: Biology (Basel). 2022 Apr 24;11(5):649. doi: 10.3390/biology11050649 (PMC9138287; doi:10.3390/biology11050649)
Supplement: Supplementary file 1 [file biology-11-00649-s001.zip › biology-1657988-supplementary.pdf]

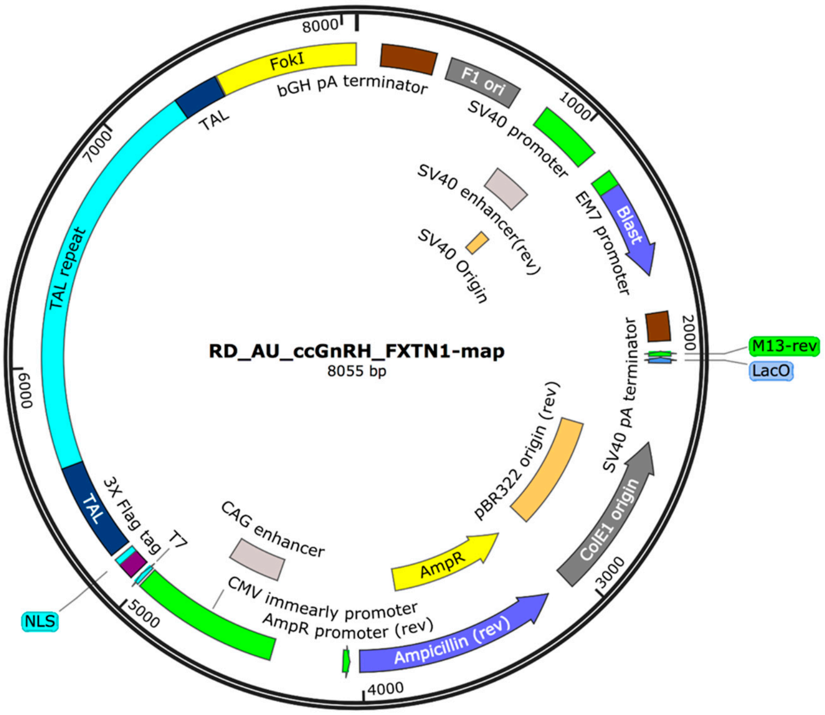

Figure S1. Schematic representation of transcription activator-like effector nucleases (TALENs) plasmid structure targeting catfish type gonadotropin-releasing hormone (cfGnRH) gene of channel catfish (*Ictalurus punctatus*).

CMV prmt cytomegalovirus promoter, *NLS* nuclear localization signal, *BGH pA* bovine growth hormone polyadenylation signal, *AmpR* ampicillin resistance gene.

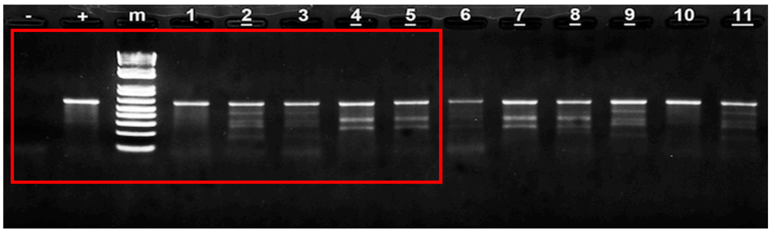

Figure S2. Original images of gels.

The result of Surveyor mutation detection in Figure 1 was shown. Red box represents the cropped area.

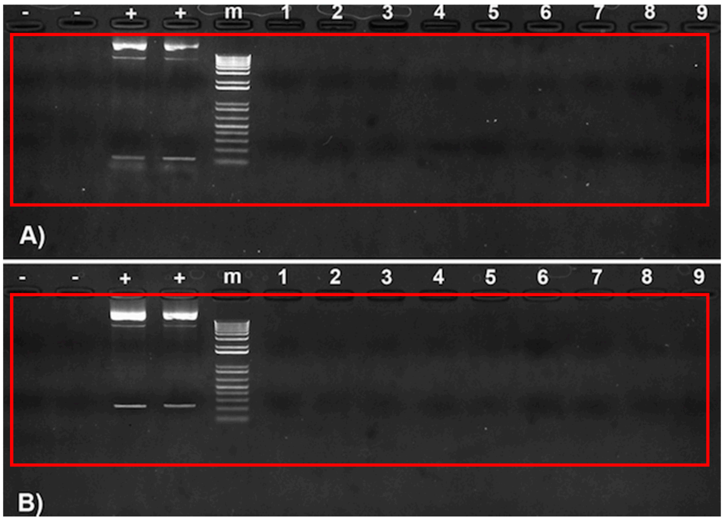

Figure S3. Original images of gels.
Results of Surveyor mutation detection in Figure 3A,B were shown. Red box represents the cropped area.

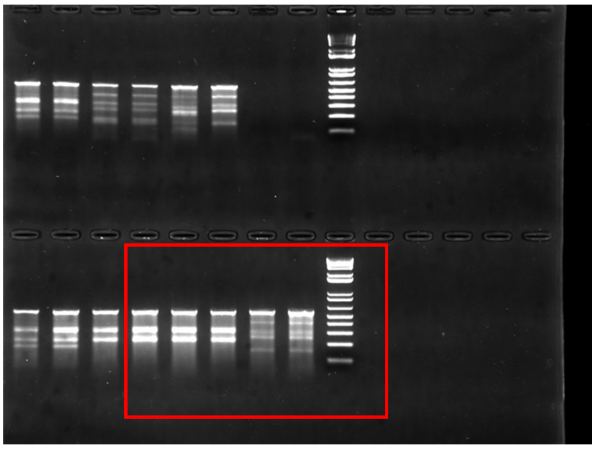

Figure S4. Original images of gels.
The results of Surveyor mutation detection in Figure 5 were shown. Red box represents the cropped area.

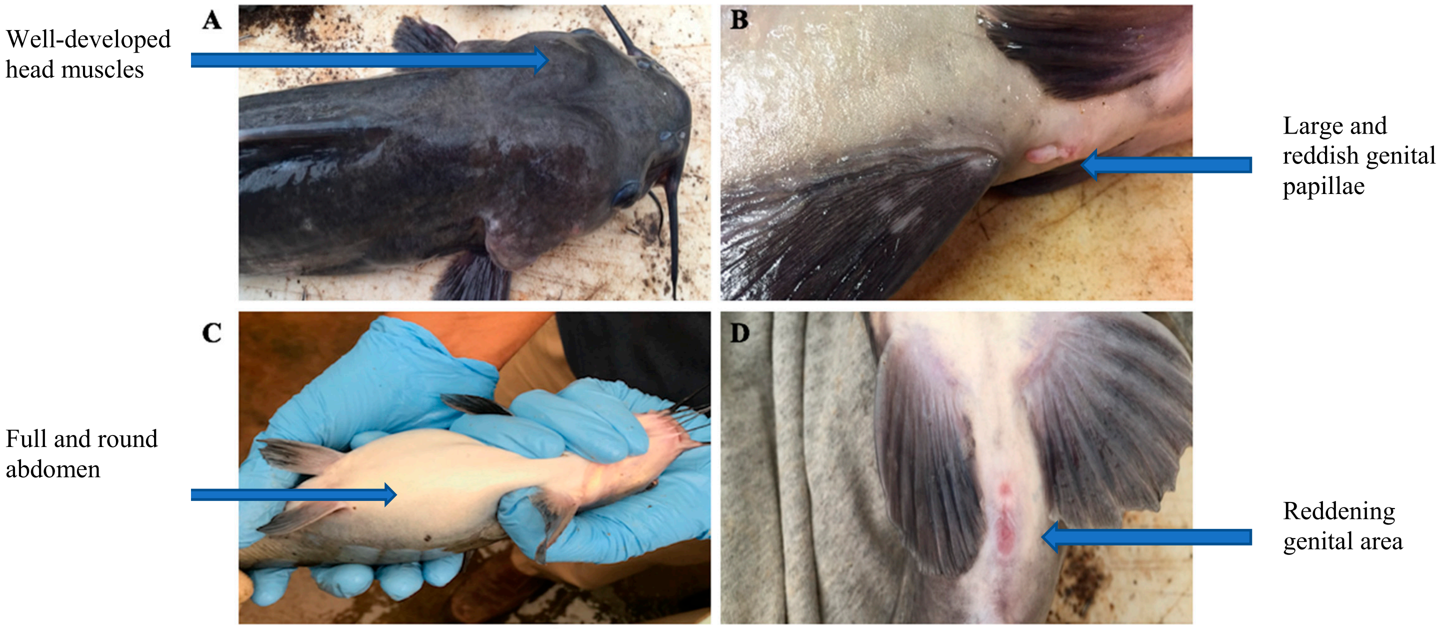

Figure S5. Images of the spawned P_1 fish with outstanding secondary sexual characteristics.
A showed the spawned male fish with well-developed head muscles.
B showed the spawned male fish with large and reddish genital papillae.
C showed the spawned female fish with full and round abdomen.
D showed the spawned female fish with reddening genital area.
